# Preventive effects of quercetin against inflammation and apoptosis in cyclophosphamide-induced testicular damage

**DOI:** 10.22038/IJBMS.2024.74458.16177

**Published:** 2024

**Authors:** Duygu Uzun-Goren, Yesim Hulya Uz

**Affiliations:** 1 Department of Histology and Embryology, Faculty of Medicine, Trakya University, Edirne, Turkey

**Keywords:** Apoptosis, Cyclophosphamide, Inflammation, Quercetin, Testis

## Abstract

**Objective(s):**

We aimed to investigate the effects of quercetin (QRC) against cyclophosphamide (CP)-induced testicular damage and how it interacts with apoptotic and inflammatory signaling pathways.

**Materials and Methods:**

Forty male Wistar rats were randomly divided into four groups, 10 in each group; Control group (corn oil, intragastrically, 14 days), QRC group (100 mg/kg QRC, dissolved in corn oil, 14 days), CP group (200 mg/kg CP, intraperitoneally, single dose on the 7th day), and CP+QRC group (100 mg/kg QRC, intragastrically, 14 days and 200 mg/kg CP, intraperitoneally, single dose on the 7th day). Animals were sacrificed one day after the last QRC application and the effects of quercetin were evaluated by histological, morphometrical, and hormonal parameters. Also, nuclear factor kappa B (NFkB), nuclear factor erythroid 2 related factor 2 (Nrf2), Bcl-2 associated X protein (Bax), and B-cell lymphoma-2 (Bcl-2) immunoreactivities were evaluated immunohistochemically.

**Results:**

CP increased the testicular weight/body weight ratio, significantly decreasing body weights and testicular weights. All hormone levels were also reduced significantly. Morphometrically, seminiferous tubules diameter and germinal epithelial thickness decreased, while a significant increase was determined in interstitial field width in addition to histological damage. Furthermore, immunohistochemical findings also indicated that NFkB and Bax immunoreactivity were increased in the CP group, whereas significant decrease was seen in Nrf2 and Bcl-2 immunoreactivity. Apoptotic cell and tubule index were reduced in CP. QRC ensured improvement in all findings.

**Conclusion:**

Data showed us, that QRC may have preventive effects in CP-induced testicular damage by acting on NFkB, Nrf2, Bax, and Bcl-2 pathways.

## Introduction

Infertility is an important problem affecting approximately 15% of couples in the world and approximately half of infertility problems result from male individuals. High and long-term use of chemotherapeutic drugs for treatment is effective in male infertility caused by testicular dysfunction (1). Cyclophosphamide is a cytotoxic alkylating agent and is commonly used in clinics for its antitumoral and immunosuppressive effects. Although it is an effective drug in the treatment, it causes damage to many tissues, including the testes, by acting against cells in the division phase in healthy tissues (2, 3). The active metabolites of CP are phosphoramide mustard and acrolein, which are involved in the effectiveness of the drug. Phosphoramide mustard is activated with a reactive intermediate that causes DNA breaks, disruptions in DNA synthesis, and eventual cell death. On the other hand, acrolein takes part in cellular toxicity by inactivating DNA repair proteins (2).

Despite the therapeutic efficacy of the drug, its side effects on many systems, including the genital system, have been indicated in many clinical and experimental studies (2, 4-6). In addition, experimental studies have also shown decreased body and testicular weights, sperm viability, number and motility, serum testosterone, follicle-stimulating hormone (FSH), and luteinizing hormone (LH) levels. Also, degenerative changes result from an interruption in the spermatogenesis process in testis tissues following CP treatment of male rats (2, 3, 7-10). Although the mechanisms of CP in testicular toxicity are still not clear, according to studies, CP acts by causing oxidative stress by disrupting the oxidation-antioxidation balance in tissues (4, 11, 12). Studies have shown that CP therapy is effective in the formation of free radicals and reactive oxygen species (ROS) and increases the formation of ROS. Increased ROS causes induction of apoptotic and inflammatory signaling pathways (1, 2, 9, 11, 13).

Nuclear factor kappa B (NFkB) functions as a regulator of the inflammatory response by inducing the transcription of proinflammatory cytokines in cells in which oxidative stress is triggered (14). Rezaei *et al.* (5) reported that immunoreactivity of NFkB in testis tissue was detected as intense staining including primary spermatocyte and Leydig cells of CP-treated mice. Nuclear factor erythroid 2 related factor 2 (Nrf2) balances antioxidant/detoxification genes and scavenges oxidative stress-induced ROS via translocating into the nucleus to interact with AREs (15). Furthermore, the evidence from studies shows that there is an interaction between Nrf2 and NFkB signaling pathways (16, 17). Maremanda *et al.* (16) reported that NFkB protein expression increased significantly due to induction of the inflammatory pathway, whereas Nrf2 expression, which is a member of the antioxidant defense system, decreased significantly in CP-induced testicular damage. Bcl-2 associated X protein (Bax) / B-cell lymphoma-2 (Bcl-2) signaling pathway plays a vital role in mitochondria-mediated apoptosis and hence impacts the development of testicular injury due to CP treatment. In animal studies, the expression of Bax increases, whereas Bcl-2 expression decreases due to CP treatment in rats (18, 19).

Phenolic compounds present as nutritional antioxidants can protect healthy tissues from oxidative-induced damage (5). Quercetin (QRC) is one of the members of the flavonoid group, which consists of phenolic components and is found in many food sources (20). QRC successfully protects cells from the degenerative effects of drug toxicity by scavenging free radicals and increasing endogenous antioxidant levels (21, 22). Researchers (23) evaluated the antioxidant activity of QRC in their experimental studies and stated that ROS induces the NFkB pathway and QRC, which is an antioxidant component, provides ROS regulation in the cell and increases Nrf2 activation through the antioxidant response element ARE. Also, in one study, it was reported that QRC reduced the expression of Bax and induced Bcl-2 in triptolide-induced apoptosis (24).

According to our literature review, no study was found that evaluated the antiapoptotic and anti-inflammatory effects of QRC against CP-induced testicular damage with changes in intracellular signaling pathways immunohistochemically. Our study was performed using histological, morphometric, immunohistochemical, and hormonal parameters with the assumption that CP causes testicular damage and QRC has a preventive effect in rats.

## Materials and Methods

***Chemicals***

Cyclophosphamide was purchased from EIP ECZACIBASI (Endoxan, Istanbul, Turkey), and QRC was obtained from Alfa Aesar (Kander, Germany).

***Ethics committee approval and animals***

The study was approved by the Experimental Local Ethics Committee of Trakya University (permission number: TUHDYEK-2017/07).

***Experimental design and animals***

Forty male *Wistar albino* rats, three months old, weighing 250–300 g were obtained from the Experimental Animals Research Unit (Trakya University, Edirne, Turkey). Throughout the experiment, animals were cared for in standard laboratory conditions (22 ± 1 °C, in a 12-hr light/dark cycle; relative humidity 55%) and were allowed unrestricted access to water and food. The rats were randomly divided into four equal groups (n=10) as follows:

Control group was given only solvent (corn oil) for 14 days intragastrically (IG), QRC group was administered i.g. QRC (100 mg/kg) dissolved in corn oil for 14 days, CP group was injected with a single dose CP (200 mg/kg) intraperitoneally (IP) on the 7th day of the study, CP+QRC group was administered i.g. QRC (100 mg/kg) for 14 days, and injected with CP (200 mg/kg) IP (2, 25).

Twenty-four hours after the last dose of QRC, all rats were sacrificed by cervical dislocation under xylazine (Rompun, Bayer, Istanbul, Turkey) and ketamine (Ketasol, Richter Pharma AG, Wels, Austria) anesthesia. Blood samples were collected by cardiac puncture and centrifuged for hormonal analysis and testis tissues were taken from the animals for histological and immunohistochemical examinations. In addition, body weights and testicular weights of animals were recorded. The ratio of both testis’s weight to body weight was also calculated.

***Serum testosterone, FSH, and LH ELISA evaluations***

In the obtained serum samples, serum testosterone, FSH, and LH were measured using Enzyme-Linked Immunosorbent Assay (ELISA (Elabscience, Houston, TX, USA)). All hormone analyses were performed according to the instructions in the datasheets.

***Morphometric and histological analysis***

All rats were sacrificed while under anesthesia and their testes were dissected and fixed with neutral 10% formalin (Sigma-Aldrich, Taufkirchen, Germany). Testes samples were processed by routine histological tissue procedure. Sections of 5 μm thickness were taken from paraffin blocks and hematoxylin and eosin (H&E) staining was performed. The preparations were examined and photographed with a light microscope (Olympus BX51, Tokyo, Japan) and an attachment (Olympus DP20 microscopic digital camera system) at different magnifications (x100, x200, x400).

In H&E stained preparations, seminiferous tubules diameter, germinal epithelial thickness, and the interstitial field width were measured using the Imaging Analysis System (Version 2.11.5.1, Kameram-Argenit, Istanbul, Turkey) at x100 or x400 magnification. For these measurements, the cross-section of 100 randomly selected round or near-round tubules was evaluated for each animal. Likewise, interstitial field widths were calculated by measuring 100 different sites. All these parameters were evaluated on 5 testes sections of each animal and 20 different fields (26, 27).

Tubular alterations were evaluated histologically, by examining 6 testes sections from each animal at x100 magnification, according to the following criteria: Detachment (rupture of spermatocyte cells from the germinal epithelium), sloughing (shedding of germ cells from the germinal epithelium into the lumen), and vacuolization (the formation of cavities in the seminiferous tubule). The mean percentage for each section was determined via the number of normal or damaged round tubules divided by the total round tubules in the same area for each sample, and the results were multiplied by 100. Three different fields were evaluated for each section and their average was taken. Evaluations were determined as in previous studies (27, 28).

***NFkB, Nrf2, Bax and Bcl-2 immunohistochemical evaluations***

Immunohistochemical staining was performed as previously reported using the streptavidin-biotin complex method (27). The slides were incubated with anti-NFkB antibody (1:100; Thermo Scientific/Lab Vision) for 1 hr at room temperature, anti-Nrf2 (1:400; Abcam, Cambridge, MA, USA), anti-Bax (1:250; Abcam), and anti-Bcl-2 (1:200; Abcam) antibodies were also incubated overnight in a humidified chamber at 4 °C. Negative controls were substituted with phosphate-buffered saline (PBS) instead of the primary antibodies. Biotinylated secondary antibody (Biotinylated Goat Anti-Polyvalent, Thermo Scientific/LabVision) and then streptavidin-peroxidase (Thermo Scientific/LabVision) were applied for 10 min at room temperature. Afterward, 3-amino-9-ethylcarbazole (AEC, Thermo Scientific/Lab Vision) was used to chromogenize and counterstain with Mayer’s hematoxylin.

The immunoreactivities of NFkB, Nrf2, Bax, and Bcl-2 were evaluated semi-quantitatively with the HSCORE method calculated as HSCORE = Σi x Pi. All scores were classified based on the percentages of stained cells for each intensity score (Pi) and the following intensity categories: (i; 0 = no staining, 1 = weak staining, 2 = moderate staining, 3 = intense staining). Five randomly selected fields were examined under the microscope at ×400 magnification by two independent observers and the obtained values were averaged (29, 30).

***TUNEL assay***

Apoptosis was determined via TUNEL assay, using the Apop Tag Plus Peroxidase In Situ Apoptosis Detection Kit (Merck Millipore, Billerica, MA, USA ApopTag). At least one hundred seminiferous tubules sections randomly selected from each specimen were evaluated for the presence of dark brown stained nucleus of apoptotic cells. The average number of TUNEL-positive cells per tubule and the number of apoptotic tubules with at least one TUNEL-positive cell were determined. The average apoptotic cell/tubule ratio (apoptotic cell index) and apoptotic tubule index were obtained for each animal (31).

***Statistical analysis***

All data were executed via SPSS version 20.0 (IBM SPSS Statistics; Armonk, NY, USA). The normal distribution of the variables was tested with the Kolmogorov-Smirnov test, and the normally distributed data were compared using one-way ANOVA followed by Tukey and Tamhane tests according to the homogeneity of the group variances. Non-normally distributed data were evaluated with the Kruskal-Wallis and then the Mann-Whitney U *post-hoc* tests. All values were written as mean ± standard deviation (SD) and the significance value was accepted as *P*<0.05.

## Results

***Body weight, testicular weight, and relative testicular weight findings***

The numerical values of body weight, testicular weight, and relative testicular weight with statistical evaluations among groups are presented in Table 1. Body and total testis weights significantly decreased in the CP group compared to the control and QRC groups (*P*<0.001 and *P*<0.01, respectively). However, in the QRC+CP group, QRC treatment significantly slowed down the decrease in body weight compared to the CP group (*P*<0.05). Total testis weight changes were slightly increased and this change was not significant. On the other hand, relative testicular weights increased in the CP group (*P*<0.001), these values were slightly decreased in the CP+QRC group compared with the CP group, but this change also was not significant.

***Serum testosterone, FSH, and LH ELISA findings***

The effect of QRC on CP-injected rats’ serum testosterone, FSH, and LH levels is presented in Table 2. All hormone levels were significantly reduced in the CP group, compared to the control and QRC groups (*P*<0.001). However, QRC treatment improved all three hormone levels significantly in the CP+QRC group compared to the CP group (*P*<0.001, *P*<0.001, and *P*<0.05 respectively; Table 2).

***Morphometric and histological findings***

Statistical comparisons of morphometric analyses between groups are presented in Table 3. While seminiferous tubules diameter and germinal epithelial thickness decreased significantly in the CP group compared to the control and QRC groups (*P*<0.001), the interstitial field width increased significantly (*P*<0.001). However, QRC contributed to testicular tissue integrity by protecting effects on all morphometric parameters in the CP+QRC group compared to CP (*P*<0.001).

**Figure 1:**
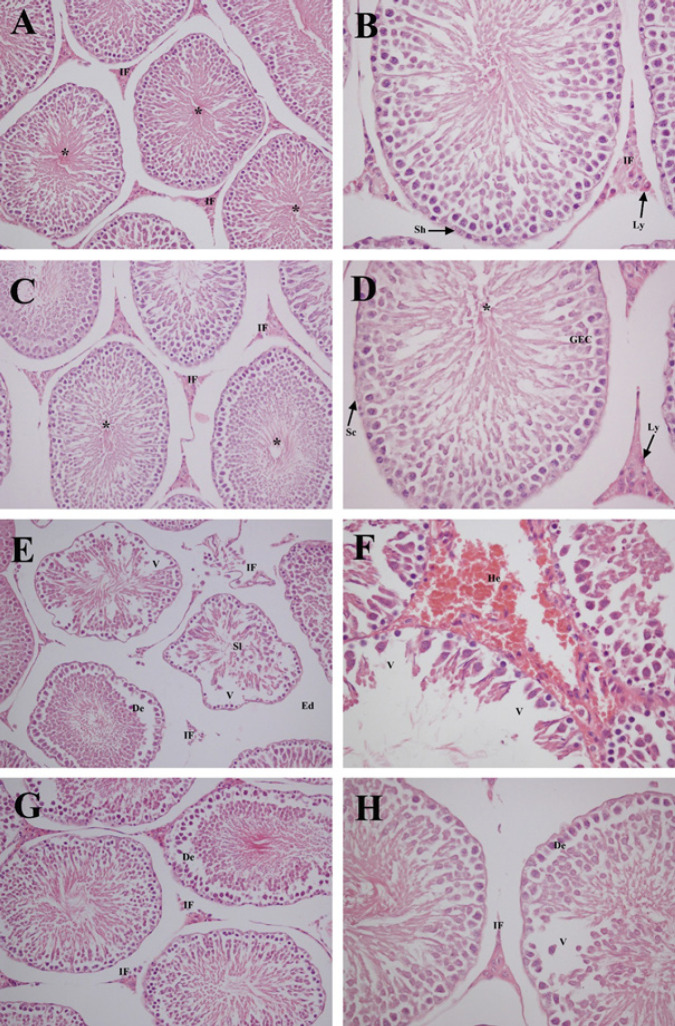
Representative photomicrographs of testis tissues in control and experimental groups of rats

Tubular histological assessments are presented in Table 4. Photomicrographs of control and QRC groups showed normal organization of mature active seminiferous tubules and germinal epithelial cells. Also, connective tissue structural elements, Leydig cells, and blood vessels were observed in normal histological structure in the interstitial field. (Figure 1. A-D). CP group revealed different levels of degenerative tubular changes, irregular arrangement of germ cells, detachment and sloughing of germinal epithelial cells, and vacuolization resulting from cell loss at germinal epithelium, giving rise to tubular atrophy (Figure 1. E and F). All degenerative tubular changes were significantly induced in the CP group compared with the control and QRC groups (*P*<0.001). Besides, irregular connective tissue, degeneration of Leydig cells, blood vessel congestion, localized hemorrhage foci, and interstitial edema were determined in the interstitial field. However, in the CP+QRC group, all histological damages were significantly decreased compared to CP-treated animals (Figure 1. G and H), with the beneficial contribution of QRC (*P*<0.001).

***Immunohistochemical findings***

The NFkB, Nrf2, Bax, and Bcl-2 HSCORE values of all groups and the statistical evaluations of these data are given in Table 5.

*NFkB immunoreactivity*

**Figure 2:**
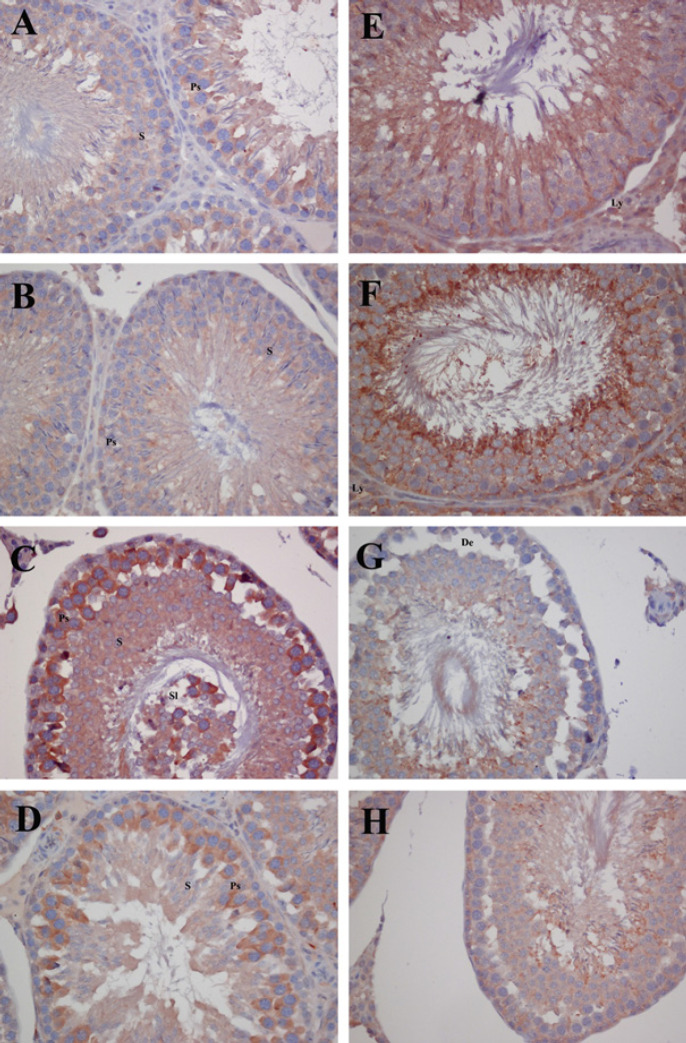
Immunohistochemical staining of NFkB and Nrf2 in the control and experimental groups of rats

The testicular immunoreactivity of NFkB was examined by immunohistochemical staining methods. NFkB immunoreactivity indicated weak staining in the germinal epithelium of control and QRC groups (Figure 2. A and B). However, in the CP group, the seminiferous tubules sections showed intense staining, especially in primary spermatocytes, and in other cells moderate or intense staining was observed. Also, in the interstitial field, Leydig cells were moderately or weakly stained (Figure 2. C). NFkB immunoreactivity was significantly increased in CP-treated testes sections compared to the control and QRC groups (*P*<0.001). With administration of QRC in addition to CP, NFkB immunoreactivity was significantly reduced compared to the CP group. (*P*<0.001, Table 5, Figure 2. D).

*Nrf2 immunoreactivity*

When Nrf2 immunoreactivity of the control and QRC groups was evaluated, it was observed to be intense or moderate staining in germinal epithelial cells, especially in spermatids near the luminal surface, in the germinal epithelium and Sertoli cells. In the interstitial field, Leydig cells were stained moderate to weak (Figure 2. E and F). However, weak staining was observed in all germinal epithelial cells in testicular sections of the CP group, and very weak staining in Leydig cells located in the interstitial field (Figure 2. G). It was determined that Nrf2 immunoreactivity in the CP group decreased significantly when compared with the control and QRC groups (*P*<0.001). In the CP+QRC group, moderate or weak staining was seen in germinal epithelial cells and Sertoli cells, while weak staining was determined in Leydig cells. In the CP+QRC group, the Nrf2 immunoreactivity was increased significantly compared to the CP group (*P*<0.001, Table 5, Figure 2. H).

*Bax and Bcl-2 immunoreactivity*

**Figure 3:**
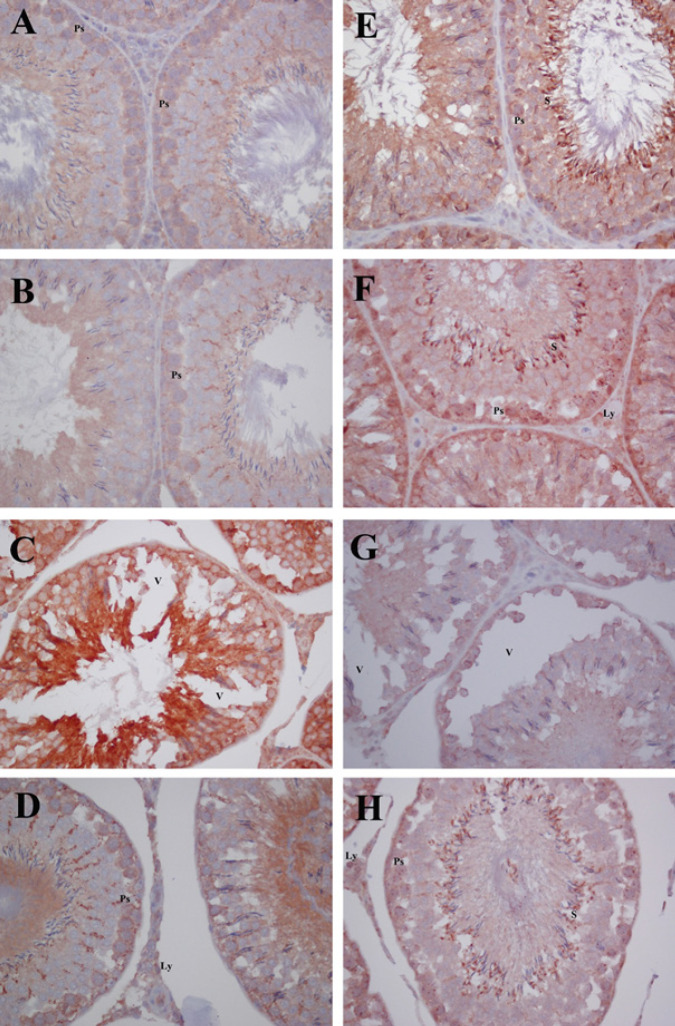
Immunohistochemical staining of Bax and Bcl-2 in the control and experimental groups of rats

For evaluating the apoptotic pathway, we examined Bax and Bcl-2 expressions in testes sections. Bax immunoreactivity was weak in the control and QRC groups. However, the CP group revealed a significant increase in Bax immunoreactivity compared to the control and QRC groups (*P*<0.001, Figure 3. A and B). In the testicular sections of the CP group, intense Bax immunoreactivity was determined in the entire germinal epithelium, more prominent in primary spermatocytes and spermatids. Between the seminiferous tubules, Leydig cells with moderate or intense staining were observed. It was noteworthy that intense Bax immunoreactivity showed perinuclear localization, especially in primary spermatocytes (Figure 3. C). Treatment with QRC prevented significantly the increase of Bax immunoreactivity in CP+QRC group of animals (*P*<0.001, Table 5, Figure 3. D).

Bcl-2 expression in testes sections was moderate in control and QRC groups, especially in primary spermatocytes and spermatids close to the tubule lumen. In addition, moderate or weak staining was seen in Leydig cells (Figure 3. E and F). In contrast to Bax, Bcl-2 immunoreactivity was decreased significantly in CP-injected animals compared to the control and QRC groups (*P*<0.001, Figure 3. G). On the other hand, QRC treated in addition to CP significantly up-regulated the immunoreactivity of Bcl-2 compared to the CP group (*P*<0.001, Table 5, Figure 3. H).

***TUNEL findings***

**Figure 4:**
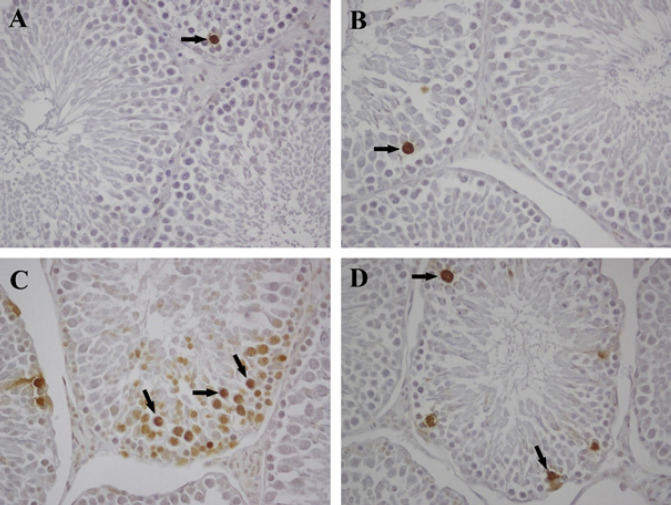
TUNEL staining of testis tissues in the control and experimental groups of rats

The TUNEL method was applied to determine apoptosis in testis tissues. The apoptotic assessment was performed by calculating the apoptotic cell index and the apoptotic tubule index. The apoptotic cell or tubule index assessment was similar in the control and QRC groups (Figure 4. A and B). However, there was a significant increase in apoptotic cell or tubule indices in the CP group compared to the control and QRC groups. (*P*<0.001, Figure 4. C). Treatment of QRC markedly reduced both the apoptotic cell or tubule indexes in the CP+QRC group compared with the CP group in testes sections (*P*<0.001, Table 6, Figure 4. D). 

**Table 1:** Comparison of body weights (BW) (g), total testicular weights (g), and testicular weight/body weight ratios (TAI = TA/VA × 100) in control and experimental groups of rats

Parameter	Control	QRC	CP	CP+QRC	*P*-value
BW change	10.1±5.17	8.7±3.5	-54.9±11.72^*^^†^	-41.4±13.61^*^^†#^	*P*<0.001
Total testicular weight	2.52±0.24	2.53±0.13	2.32±0.13^**^^††^	2.38±0.16^††^	*P*<0.05
TAI = TA/VA × 100	0.95±0.1	0.94±0.09	1.05±0.06^**^^††^	1.01±0.08	*P*<0.05

Data are expressed as mean±SD, n=10 in each group

* *P*<0.001 compared to control group; ** *P*<0.05 compared to control group, † *P*<0.001 compared to QRC group; †† *P*<0.05 compared to QRC group, # *P*<0.05 compared to CP group

CP: Cyclophosphamide; QRC: Quercetin

**Table 2:** Comparison of serum testosterone, FSH, and LH levels in control and experimental groups of rats

Parameter	Control	QRC	CP	CP+QRC	*P*-value
Testosterone (ng/ml)	9.47±4.29	9.85±5.96	0.75±0.13^*^^†^	1.76±0.58^*^^†#^	*P*<0.001
FSH (ng/ml)	20.52±10.91	22.83±8.98	0.63±0.2^*^^†^	1.92±1.03^*^^†#^	*P*<0.001
LH (mIU/ml)	56.42±19.79	57.89±16.83	0.7±0.16^*^^†^	1.49±0.96^*^^†##^	*P*<0.001

Data are expressed as mean±SD, n=10 in each group

* *P*<0.001 compared to control group, † *P*<0.001 compared to QRC group, # *P*<0.001 compared to CP group; ## *P*<0.05 compared to CP group

FSH: Follicle-stimulating hormone; LH: Luteinizing hormone; CP: Cyclophosphamide; QRC: Quercetin

**Table 3:** Comparison of seminiferous tubules diameter (µm), germinal epithelial thickness (µm), and interstitial field width (µm) in control and experimental groups of rats

Parameter	Control	QRC	CP	CP+QRC	*P*-value
Seminiferous tubules diameter	288.69±2,25	289.98±7,25	237.06±5,79^*^^†^	263.88±7,47^*^^†#^	*P*<0.001
Germinal epithelial thickness	61.51±2.14	64.52±4.24	37.45±2.4^*^^†^	53.3±2.05^*^^†#^	*P*<0.001
Interstitial field width	19.41±2.23	20.3±2.55	56.49±7.71^*^^†^	39.15±4.31^*^^†#^	*P*<0.001

Data are expressed as mean±SD, n=10 in each group

* *P*<0.001 compared to control group, † *P*<0.001 compared to the QRC group, # *P*<0.001 compared to the CP group

CP: Cyclophosphamide; QRC: Quercetin

**Table 4:** Comparison of histological structure changes of seminiferous tubules in control and experimental groups of rats

Percentage of seminiferous tubules	Control	QRC	CP	CP+QRC	*P*-value
Normal	90.45±1,94	91.28±2,67	31.46±2.63^*†^	68.93±2.178^*†#^	*P*<0.001
Detached	5.36±1.63	5.42±1.79	28.87±4.79^*†^	13.96±3.54^*†#^	*P*<0.001
Sloughed	2.06±1.29	1.17±0.70	21.59±7.25^*†^	7.48±1.72^*†#^	*P*<0.001
Vacuolized	2.13±0.73	2.12±1	27.93±3.44^*†^	15.38±2.32^*†#^	*P*<0.001

Data are expressed as mean±SD, n=10 in each group

* *P*<0.001 compared to control group, † *P*<0.001 compared to the QRC group, # *P*<0.001 compared to the CP group

CP: Cyclophosphamide; QRC: Quercetin

[A and B] the control group showed normal histological structure of testis seminiferous tubules (asterisks) and interstitial field (IF), normal seminiferous tubules, regularly organized germinal epithelial cells (GEC) and Sertoli cells (Sc), and Leydig cells interstitial tissues (IF). [C and D] In the QRC group normal histological structure was observed similar to the control group. [E and F] CP group demonstrated damaged seminiferous tubules including detachment (De), sloughing (Sl), and vacuolization (V). Also, interstitial edema (Ed), disintegration of Leydig cells (Ly), and congestion or hemorrhage (He) were seen in the interstitial field (IF). [G and H] CP+QRC group showed mild degeneration in the seminiferous tubules and interstitial field. Furthermore, seminiferous tubules with preserved structural integrity, active spermatogenesis, and relatively regular interstitial field (IF) with Leydig cells were seen in this group. However, some seminiferous tubules including detachment (De), sloughing, or vacuolization (V) were seen in some regions. Staining and magnification of photomicrographs of rat testis tissue sections [H&E x200; A, C, E, G] and [H&E x400; B, D, F, H].

CP: Cyclophosphamide; QRC: Quercetin

**Table 5:** Comparison of NFkB, Nrf2, Bax, and Bcl-2 immunoreactivities (HSCORE) in control and experimental groups of rats

Parameter	Control	QRC	CP	CP+QRC	*p*-value
NFkB	110±14.91	89±17.13	210.50±22.29^*†^	154±17.61^*†#^	*P*<0.001
Nrf2	160.50±9.85	173±11.60	101±12.20^*†^	136.50±10.55^*†#^	*P*<0.001
Bax	106±11.74	102±10.33	205±17.32^*†^	150±19^*†#^	*P*<0.001
Bcl-2	169.50±13.63	170±16.83	96.50±11.07^*†^	145±18.63^*†#^	*P*<0.001

Data are expressed as mean±SD, n=10 in each group

* *P*<0.001 compared to control group, † *P*<0.001 compared to QRC group, # *P*<0.001 compared to CP group

NFkB: Nuclear factor kappa B; Nrf2: Nuclear factor erythroid 2 related factor 2; CP: Cyclophosphamide; QRC: Quercetin; Bcl-2: B-cell lymphoma-2

[A and B] NFkB immunoreactivity was weak in the control and QRC groups. [C] Whereas, NFkB immunoreactivity increased in the CP group compared to the control and QRC groups. [D] In the CP+QRC group, NFkB immunoreactivity was significantly decreased compared to the CP group. [E and F] Nrf2 immunoreactivity was seen as moderate or intense staining in the control and QRC groups similarly. [G] However, Nrf2 immunoreactivity significantly decreased in the CP group compared to the control and QRC groups. [H] In the CP+QRC group, Nrf2 immunoreactivity was significantly increased compared to the CP group. PS, primary spermatocyte; S, spermatid; V, vacuole; Ly, Leydig cell; De, detachment; Sl, sloughing. Hematoxylin counterstain, original magnification x400 [NFkB immunoreactivity A, B, C, D] [Nrf2 immunoreactivity E, F, G, H].

NFkB: Nuclear factor kappa B; Nrf2: Nuclear factor erythroid 2 related factor 2; CP: Cyclophosphamide; QRC: Quercetin

[A and B] Bax immunoreactivity was weak in the control and QRC groups. [C] However, in the CP group, Bax immunoreactivity increased compared to the control and QRC groups. Intense Bax immunoreactivity showed perinuclear localization, especially in primary spermatocytes. [D] In the CP+QRC group, Bax immunoreactivity was significantly decreased. [E and F] Bcl-2 immunoreactivity was observed as moderate or intense and stained similarly in the control and QRC groups. [G] Whereas, Bcl-2 immunoreactivity significantly decreased in the CP group compared to the control and QRC groups. [H] In the CP+QRC group, Bcl-2 immunoreactivity was significantly increased. PS, primary spermatocyte; S, spermatid; V, vacuole; Ly, Leydig cell. Hematoxylin counterstain, original magnification x400 [Bax immunoreactivity A, B, C, D] [Bcl-2 immunoreactivity E, F, G, H].

CP: Cyclophosphamide; QRC: Quercetin; Bcl-2: B-cell lymphoma-2; Bax: Bcl-2 associated X protein

[A and B] In the control and QRC groups, a few TUNEL-positive cells (arrows) were observed in the seminiferous tubules of testis tissues. [C] The TUNEL-positive cells were significantly higher in the seminiferous tubules of testis tissues in the CP group. [D] Treatment of QRC markedly decreased the TUNEL-positive cells in the CP+QRC group. Hematoxylin counterstain, original magnification x400.

CP: Cyclophosphamide; QRC: Quercetin

**Table 6:** Comparison of apoptotic cell and apoptotic tubule indexes in control and experimental groups of rats

Parameter	Control	QRC	CP	CP+QRC	*P*-value
Apoptotic cell index	0.23±0.88	0.13±0.73	5.43±1.37^*†^	1.17±0.36^**††#^	*P*<0.001
Apoptotic tubule index	13.32±1.61	10.13±3.94	59.06±6.35^*†^	34.18±10.41^*†#^	*P*<0.001

Data are expressed as mean±SD, n=10 in each group

* *P*<0.001 compared to control group; ** *P*<0.05 compared to control group, † *P*<0.001 compared to QRC group; ††*P*<0.05 compared to QRC group, # *P*<0.001 compared to CP group

CP: Cyclophosphamide; QRC: Quercetin

## Discussion

In the present study, the preventive effect of QRC, which is a powerful antioxidant, was investigated using hormonal, histological, and immunohistochemical parameters to contribute to the prevention/reduction of testicular damage and related infertility due to CP. In addition, the anti-apoptotic and anti-inflammatory effects of QRC in testicular damage induced by CP in Wistar rats was studied to identify novel regimens for improving CP treatment. According to our findings, the decrease in body weight, serum testosterone, FSH, and LH hormone levels was significantly prevented by QRC, while damaged seminiferous tubules and interstitial fields were significantly regressed. Furthermore, QRC significantly reversed the increase of NFkB and Bax immunoreactivities and the decrease of Nrf2 and Bcl-2 immunoreactivities in CP-induced testicular damage. In addition, QRC regressed CP-induced DNA fragmentation in the testis. To our knowledge, this is the first study to investigate the effects of QRC on CP-induced testicular damage through immunohistochemical examinations of apoptotic and inflammatory signaling pathways in an experimental model.

Cyclophosphamide is an effective cytotoxic alkylating agent in the treatment of hematological and solid tumors as well as many neoplastic and autoimmune diseases and is used immunosuppressive in organ transplantation (9, 10, 12). Although extensive used, it is also associated with some serious side effects, such as testicular toxicity and infertility (5, 7, 12). The toxic effect of CP may be mediated by disrupting the oxidation-antioxidation balance system in testes (9, 11, 13, 32).

Numerous nutritional antioxidants and dietary supplements have been utilized to deal with testicular toxicity caused by CP in experimental models, and many of them report encouraging results (10-12, 33-35).

Similar to previous studies on CP toxicity, a significant decrease was observed in body weight and total testicular weight in our study (2, 9, 36). In addition, studies showing a significant increase in testicular weight/body weight ratios are also consistent with our findings (36,37). In the study conducted by Kim *et al.* (37) 150 mg/kg of CP was given intraperitoneally for 2 consecutive days and it was determined that the food consumption of the animals that received CP injection also decreased significantly. It seems that the body weight loss and the decrease in food consumption seen in the CP group are associated with dose-dependent CP toxicity. Therefore the testicular weight/body weight ratio was found to be higher in the CP group. Besides, the cytotoxic effect of CP may have caused atrophy in the testicular tissue and significantly reduced testicular weights. With QRC, body weight loss was less significant in the CP+QRC group compared to CP. Testicular weights were slightly preserved in the CP+QRC group, but it was not statistically significant. 

Another finding associated with testicular damage is a disruption in the production of serum testosterone, FSH, and LH hormones. In our study, there was a significant decrease in all hormone levels in the CP group compared to the control and QRC groups, which is consistent with previous studies (2, 4, 38, 39). According to previous studies, CP may have a detrimental effect on the pituitary gland, which plays a pivotal role in the secretion of these hormones, and the decrease in serum testosterone may be due to CP-induced membrane lipid peroxidation and Leydig cell degeneration in testes (33, 40). QRC treatment significantly increased all three hormone levels compared to the CP. In addition, QRC significantly increased these hormones in lead-induced testicular toxicity (41).

CP is cytotoxic to cells with high mitotic activity, therefore, it makes the testicular and germinal epithelium, which has a high proliferative rate, a target for the detrimental effects of the drug. In our study, we evaluated the histological changes in the seminiferous tubules. In the CP group, the number of normal seminiferous tubules was significantly decreased compared to the control and QRC groups, whereas, detached, sloughed, and vacuolized damaged seminiferous tubules were increased significantly. Disorganization and tubular atrophy in the seminiferous tubules, separation of the primary spermatocytes from the germinal epithelium, vacuolization, immature cell sloughing, and congestion or hemorrhage accompanied by edema in the interstitial field were observed. Degenerative changes seen in seminiferous tubules in CP exposure are in agreement with the literature (36,40,42). In our study, the deterioration in morphometric data in the CP group suggests that testicular damage could be linked to the observed significant histological changes. As a result of our literature review, our findings were consistent with other CP studies (39, 40, 43). Besides, QRC significantly decreased the number of detached, sloughed, and vacuolized seminiferous tubules compared to the CP group. Positive changes were also observed in morphometric data depending on the histological changes. Also, it was reported that seminiferous tubules diameters and germinal epithelial thicknesses increased, while the interstitial field width decreased significantly when 50 mg/kg QRC was given daily together with arsenic administration, which causes testicular damage by inducing oxidative stress (44). Our findings were parallel to this study. We think that CP-induced ROS production and increase in oxidative stress may have degenerative effects on seminiferous tubules and decreased seminiferous tubules diameter and germinal epithelial thickness by causing Leydig cell degeneration by damaging DNA, protein, and enzymes, and indirectly impairing steroidogenesis and spermatogenesis. Consequently, this degeneration may be due to increased edema in the interstitial field between the tubules with reduced tubular diameter as a result of atrophy of the tubules. QRC may contribute to the preservation of histological structure by reducing ROS production, maintaining mitogenic activities of germinal epithelial cells, and maintaining testosterone production in Leydig cells. 

NFkB is a transcription factor that acts on the expression of many proinflammatory genes such as cytokines, chemokines, and adhesion molecules, and initiates the inflammatory pathway (14). In the present study, NFkB immunoreactivity was significantly increased in the CP group, whereas it significantly decreased in the CP+QRC group compared to the CP group. Increased NFkB immunoreactivity in CP-induced testicular damage has been previously demonstrated (5, 16). It has also been reported that QRC causes a decrease in NFKB expression in experimental toxicity studies with different agents (45-47). In our study, we think that QRC may have anti-inflammatory effects on germinal epithelial cells by suppressing NFKB expression, and may have contributed positively to the steroidogenesis in Leydig cells and spermatogenesis in the seminiferous tubules.

 The transcription factor Nrf2 regulates the recruitment of inflammatory cells and regulates gene expression through the antioxidant response element ARE. In addition, Nrf2 is responsible for the regulation of antioxidant enzyme expressions, which have been examined in many biochemical studies (15, 48). In this study, while Nrf2 immunoreactivity was significantly decreased in the CP group compared with the control and QRC groups, it was significantly increased in the CP+QRC group compared with the CP group. CP administration causes Nrf2 to be unable to translocate to the nucleus due to disruption in its phosphorylation, resulting in an inability to produce antioxidant enzymes and tissue damage (49). According to studies, it has been reported that Nrf2 activates the antioxidant defense system by antagonizing NFkB activity (50, 51). Similar to our findings, Maremanda *et al.* (16) reported that NFkB protein expression increased significantly in testicular damage due to CP, whereas Nrf2 expression decreased significantly. Several studies have shown that QRC reduces drug-induced oxidative stress by increasing Nrf2 activation and blocking NFkB signaling (48, 52). Accordingly, the antagonist relationship between them shows that Nrf2 and NFkB pathways regulate the intracellular redox balance and the response of cells to stress and inflammation. The data obtained in our study support this idea.

The Bcl-2 family is an important cell death regulator that controls apoptosis in cells (53). Bax and Bcl-2 are members of the Bcl-2 protein family, and the balance between these two proteins determines the survival or death of cells following an apoptotic stimulus. Bax triggers the release of cytochrome c and thus counteracts the cytoprotective effect of Bcl-2. On the other hand, Bcl-2 works to inhibit cytochrome c transition from mitochondria to cytosol (54). In our study, a significant increase was determined in Bax immunoreactivity, while Bcl-2 protein showed a significant decrease in the CP group similar to other studies (55, 56). However, in the CP+QRC group, Bax protein significantly decreased, contrary to Bcl-2, immunoreactivity increased significantly compared to the CP group. In many studies, in which gene and protein expressions and immunohistochemical evaluations of Bax and Bcl-2 proteins in CP-induced testicular toxicity were performed, results consistent with our findings were obtained (2, 24, 32). In addition, Bax immunoreactivity was weakly diffuse and cytoplasmic in the control and QRC groups, while it was intensely and occasionally perinuclear localized in the CP group. The translocation and concentration of cytosolic Bax in the perinuclear area in the cell is a sign that Bax plays a role in inducing apoptosis.

To support the relationship between Bax and Bcl-2, we evaluated apoptotic cell and tubule indexes by the TUNEL method in our study. Apoptotic cell and apoptotic tubule indexes of the CP group were significantly increased compared to the control and QRC groups. However, these values were significantly decreased in the CP+QRC group compared to the CP group. In different experimental studies in which oxidative stress was induced and apoptosis increased, it was reported that QRC significantly suppressed the induced apoptotic pathway (57-59). According to our findings and current studies, it can be stated that QRC prevents or reduces DNA fragmentation by preventing the harmful effects of ROS in germinal epithelial cells.

Our research is the first study to declare the preventive effect of QRC on CP-induced testicular tissue damage in terms of apoptotic and inflammatory intracellular signaling pathways and effective transcription factors via evaluating hormone assays, tissue histology, and immunohistochemistry. In our study, CP induced testicular damage and associated with the occurrence of this damage, hormonal imbalance, increase in intracellular NFkB and Bax immunoreactivities, decrease in Nrf2 and Bcl-2 immunoreactivities and increase in apoptotic cell or tubule indexes were observed. QRC, a powerful antioxidant, anti-apoptotic, and anti-inflammatory phenolic component, contributed to improving all parameters compared to the toxic effects of CP.

## Conclusion

We think that QRC has a preventive role against CP-induced testicular damage by regulating apoptotic and inflammatory pathways, such as decreased NFkB and Bax immunoreactivities and increased Nrf2 and Bcl-2 immunoreactivities. Therefore, we believe that QRC may be effective in the treatment of infertility as a result of CP-induced testicular damage in clinical practice.

## Authors’ Contributions

D UG and YH U designed the experiments; D UG performed experiments, data collection, data analysis, protocol development, and manuscript writing; YH U managed/organized the research work, conceived the study, made critical revisions, finalized the manuscript, and managed the manuscript submission. All authors contributed to the article and approved the submitted version.

## Conflicts of Interest

No conflicts of interest are declared by the authors.

## References

[1] Zhao H, Jin B, Zhang X, Cui Y, Sun D, Gao C et al (2015). Yangjing capsule ameliorates spermatogenesis in male mice exposed to cyclophosphamide. Evid Based Complement Alternat Med.

[2] Abd El Tawab AM, Shahin NN, Abdel Mohsen MM (2014). Protective effect of Satureja montana extract on cyclophosphamide-induced testicular injury in rats. Chem Biol Interact.

[3] Şekeroğlu V, Aydın B, Şekeroğlu ZA, Viscum album L (2011). extract and quercetin reduce cyclophosphamide-induced cardiotoxicity, urotoxicity and genotoxicity in mice. Asian Pacific J Cancer Prev.

[4] Ebokaiwe AP, Obasi DO, Njoku RC, Osawe S (2022). Cyclophosphamide-induced testicular oxidative-inflammatory injury is accompanied by altered immunosuppressive indoleamine 2, 3-dioxygenase in wister rats: influence of dietary quercetin. Andrologia.

[5] Rezaei S, Hosseinimehr SJ, Zargari M, Karimpour Malekshah A, Mirzaei M, Talebpour Amiri F (2021). Protective effects of sinapic acid against cyclophosphamide-induced testicular toxicity via inhibiting oxidative stress, caspase-3 and NF-kB activity in BALB/c mice. Andrologia.

[6] Blumenfeld Z, von Wolff M (2008). GnRH-analogues and oral contraceptives for fertility preservation in women during chemotherapy. Hum Reprod Update.

[7] Adana MY, Imam A, Bello AA, Sunmonu OE, Alege EP, Onigbolabi OG (2022). Oral thymoquinone modulates cyclophosphamide-induced testicular toxicity in adolescent Wistar rats. Andrologia.

[8] Cao Y, Wang X, Li S, Wang H, Yu L, Wang P (2017). The effects of L-carnitine against cyclophosphamide-induced injuries in mouse testis. Basic Clin Pharmacol Toxicol.

[9] Jalali AS, Hasanzadeh S, Malekinejad H (2012). Crataegus monogyna aqueous extract ameliorates cyclophosphamide-induced toxicity in rat testis: stereological evidences. Acta Med Iran.

[10] Lu WP, Mei XT, Wang Y, Zheng YP, Xue YF, Xu DH (2015). Zn(II) curcumin protects against oxidative stress, deleterious changes in sperm parameters and histological alterations in a male mouse model of cyclophosphamide-induced reproductive damage. Environ Toxicol Pharmacol.

[11] Kaya C, Barbaros Baseskioglu A, Yigitaslan S, Yasemin Ozatik F, Ozatik O, Uslu S (2019). The therapeutic potential of amifostine on cyclophosphamide-induced testicular dysfunction in rats: an experimental study. Int J Reprod Biomed.

[12] Parandin R, Ghowsi M, Dadbod A (2023). Protective effects of hydroalcoholic extract of Rosa canina  fruit on cyclophosphamide-induced testicular toxicity in mice. Avicenna J Phytomed.

[13] Liu F, Li XL, Lin T, He DW, Wei GH, Liu JH (2012). The cyclophosphamide metabolite, acrolein, induces cytoskeletal changes and oxidative stress in sertoli cells. Mol Biol Rep.

[14] Lawrence T (2009). The nuclear factor NF-kappaB pathway in inflammation. Cold Spring Harb Perspect Biol.

[15] Tu W, Wang H, Li S, Liu Q, Sha H (2019). The anti-inflammatory and anti-oxidant mechanisms of the keap1/Nrf2/ARE signaling pathway in chronic diseases. Aging Dis.

[16] Maremanda KP, Khan S, Jena G (2014). Zinc protects cyclophosphamide-induced testicular damage in rat: involvement of metallothionein, tesmin and Nrf2. Biochem Biophys Res Commun.

[17] Li W, Khor TO, Xu C, Shen G, Jeong WS, Yu S (2008). Activation of Nrf2-antioxidant signaling attenuates NFkappaB-inflammatory response and elicits apoptosis. Biochem Pharmacol.

[18] Koohsari M, Ahangar N, Mohammadi E, Talebpour Amiri F, Shaki F (2020). Effects of tramadol administration on male reproductive toxicity in wistar rats the role of oxidative stress, mitochondrial dysfunction, apoptosis-related gene expression, and nuclear factor kappa B signalling. Bratisl Lek Listy.

[19] Yuan D, Wang H, He H, Jia L, He Y, Wang T (2014). Protective effects of total flavonoids from epimedium on the male mouse reproductive system against cyclophosphamide-induced oxidative injury by up-regulating the expressions of SOD3 and GPX1. Phytother Res.

[20] Liu X, Song L (2022). Quercetin protects human liver cells from o,p›-DDT-induced toxicity by suppressing Nrf2 and NADPH oxidase-regulated ROS production. Food Chem Toxicol.

[21] Onur M, Yalçın E, Çavuşoğlu K, Acar A (2023). Elucidating the toxicity mechanism of AFM2 and the protective role of quercetin in albino mice. Sci Rep.

[22] Alizadeh SR, Ebrahimzadeh MA (2022). Quercetin derivatives: drug design, development, and biological activities, a review. Eur J Med Chem.

[23] Xu D, Hu MJ, Wang YQ, Cui YL (2019). Antioxidant activities of quercetin and its complexes for medical application. Molecules.

[24] Hu J, Yu Q, Zhao F, Ji J, Jiang Z, Chen X (2015). Protection of quercetin against triptolide-induced apoptosis by suppressing oxidative stress in rat leydig cells. Chemi Biol Interact.

[25] Şengül E, Gelen V, Gedikli S, Özkanlar S, Gür C, Çelebi F (2017). The protective effect of quercetin on cyclophosphamide-Induced lung toxicity in rats. Biomed Pharmacother.

[26] Nouri HS, Azarmi Y, Movahedin M (2009). Effect of growth hormone on testicular dysfunction induced by methotrexate in rats. Andrologia.

[27] Delen O, Uz YH (2021). Protective effect of pyrrolidine dithiocarbamate against methotrexate-induced testicular damage. Hum Exp Toxicol.

[28] Orazizadeh M, Khorsandi L, Absalan F, Hashemitabar M, Daneshi E (2014). Effect of beta-carotene on titanium oxide nanoparticles-induced testicular toxicity in mice. J Assist Reprod Genet.

[29] Uz YH, Murk W, Yetkin CE, Kayisli UA, Arici A (2010). Expression and role of interleukin-23 in human endometrium throughout the menstrual cycle and early pregnancy. J Reprod Immunol.

[30] Uzun-Goren D, Uz YH (2022). Protective effect of curcumin against gentamicin-induced nephrotoxicity mediated by p38 MAPK, nuclear factor- kappa B, nuclear factor erythroid 2-related factor 2. Iran J Kidney Dis.

[31] Yazdani I, Majdani R, Ghasemnejad-Berenji M, Dehpour AR (2019). Comparison of multiple doses of cyclosporine A on germ cell apoptosis and epididymal sperm parameters after testicular ischemia/reperfusion in rats. Exp Mol Pathol.

[32] Umamaheswari S, Girish C, Basu D (2022). Effects of cleistanthus collinus on the reproductive system of male wistar rats. JBRA Assist Reprod.

[33] Oyagbemi AA, Omobowale TO, Saba AB, Adedara IA, Olowu ER, Akinrinde AS (2016). Gallic acid protects against cyclophosphamide-induced toxicity in testis and epididymis of rats. Andrologia.

[34] Can S, Çetik Yıldız S, Keskin C, Şahintürk V, Cengiz M, Appak Başköy S (2022). Investigation into the protective effects of Hypericum triquetrifolium turra seed against cyclophosphamide-induced testicular injury in sprague dawley rats. Drug Chem Toxicol.

[35] Ebokaiwe AP, Ushang OR, Ogunwa TH, Kikiowo B, Olusanya O (2022). Quercetin attenuates cyclophosphamide induced-immunosuppressive indoleamine 2,3-dioxygenase in the hippocampus and cerebral cortex of male wister rats. J Biochem Mol Toxicol.

[36] Motawi TM, Sadik NA, Refaat A (2010). Cytoprotective effects of DL-alpha-lipoic acid or squalene on cyclophosphamide-induced oxidative injury: an experimental study on rat myocardium, testicles and urinary bladder. Food Chem Toxicol.

[37] Kim SH, Lee IC, Ko JW, Shin IS, Moon C, Kim SH (2016). Mechanism of protection by diallyl disulfide against cyclophosphamide-induced spermatotoxicity and oxidative stress in rats. Mol Cell Toxicol.

[38] Ekeleme-Egedigwe CA, Famurewa AC, David EE, Eleazu CO, Egedigwe UO (2019). Antioxidant potential of garlic oil supplementation prevents cyclophosphamide-induced oxidative testicular damage and endocrine depletion in rats. J Nutr Intermed Metab.

[39] Hamzeh M, Hosseinimehr SJ, Karimpour A, Mohammadi HR, Khalatbary AR, Talebpour Amiri F (2019). Cerium oxide nanoparticles protect cyclophosphamide-induced testicular toxicity in mice. Int J Prev Med.

[40] Anan HH, Zidan RA, Abd El-Baset SA, Ali MM (2018). Ameliorative effect of zinc oxide nanoparticles on cyclophosphamide induced testicular injury in adult rat. Tissue Cell.

[41] Al-Omair MA, Sedky A, Ali A, Elsawy H (2017). Ameliorative potentials of quercetin against lead-induced hematological and testicular alterations in albino rats. Chin J Physiol.

[42] Mohammadi F, Nikzad H, Taghizadeh M, Taherian A, Azami-Tameh A, Hosseini SM (2014). Protective effect of zingiber officinale extract on rat testis after cyclophosphamide treatment. Andrologia.

[43] Hosseini A, Zare S, Borzouei Z, Ghaderi Pakdel F (2018). Cyclophosphamide-induced testicular toxicity ameliorate by American ginseng treatment: an experimental study. Int J Reprod Biomed.

[44] Jahan S, Ain QU, Ullah H (2016). Therapeutic effects of quercetin against bisphenol A induced testicular damage in male sprague dawley rats. Syst Biol Reprod Med.

[45] Fadda LM, Attia HA, Al-Rasheed NM, Ali HM, Al-Rasheed NM (2018). Roles of some antioxidants in modulation of cardiac myopathy induced by sodium nitrite via down-regulation of mRNA expression of NF-κB, Bax, and flt-1 and suppressing DNA damage. Saudi Pharm J.

[46] Ebokaiwe AP, Mathur PP, Farombi EO (2016). Quercetin and vitamin E attenuate bonny light crude oil-induced alterations in testicular apoptosis, stress proteins and steroidogenic acute regulatory protein in wistar rats. Drug Chem Toxicol.

[47] Abarikwu SO, Pant AB, Farombi EO (2013). Quercetin decreases steroidogenic enzyme activity, NF-κB expression, and oxidative stress in cultured leydig cells exposed to atrazine. Mol Cell Biochem.

[48] Sanjay S, Girish C, Toi PC, Bobby Z (2021). Quercetin modulates NRF2 and NF-κB/TLR-4 pathways to protect against isoniazid- and rifampicin-induced hepatotoxicity in vivo. Can J Physiol Pharmacol..

[49] Le X, Luo P, Gu Y, Tao Y, Liu H (2015). Squid ink polysaccharide reduces cyclophosphamide-induced testicular damage via Nrf2/ARE activation pathway in mice. Iran J Basic Med Sci.

[50] Wardyn JD, Ponsford AH, Sanderson CM (2015). Dissecting molecular cross-talk between Nrf2 and NF-κB response pathways. Biochem Soc Trans.

[51] Sivandzade F, Alqahtani F, Sifat A, Cucullo L (2020). The cerebrovascular and neurological impact of chronic smoking on post-traumatic brain injury outcome and recovery: An in vivo study. J Neuroinflammation.

[52] Yardim A, Kandemir FM, Ozdemir S, Kucukler S, Comakli S, Gur C (2020). Quercetin provides protection against the peripheral nerve damage caused by vincristine in rats by suppressing caspase 3, NF-κB, ATF-6 pathways and activating Nrf2, Akt pathways. Neurotoxicology.

[53] Kale J, Osterlund EJ, Andrews DW (2018). BCL-2 family proteins: Changing partners in the dance towards death. Cell Death Differ.

[54] Wang Q, Zhao XF, Ji YL, Wang H, Liu P, Zhang C (2010). Mitochondrial signaling pathway is also involved in bisphenol a induced germ cell apoptosis in testes. Toxicol Lett.

[55] Mehrbod P, Ande SR, Alizadeh J, Rahimizadeh S, Shariati A, Malek H et al (2019). The roles of apoptosis, autophagy and unfolded protein response in arbovirus, influenza virus, and HIV infections. Virulence.

[56] Adams JM, Cory S (2007). The Bcl-2 apoptotic switch in cancer development and therapy. Oncogene.

[57] Khodabandeh Z, Dolati P, Zamiri MJ, Mehrabani D, Bordbar H, Alaee S (2021). Protective effect of quercetin on testis structure and apoptosis against lead acetate toxicity: an stereological study. Biol Trace Elem Res.

[58] Habas K, Brinkworth MH, Anderson D (2017). Diethylstilbestrol induces oxidative DNA damage, resulting in apoptosis of spermatogonial stem cells in vitro. Toxicology.

[59] Wang JY, Nie YX, Dong BZ, Cai ZC, Zeng XK, Du L (2021). Quercetin protects islet β-cells from oxidation-induced apoptosis via Sirt3 in T2DM. Iran J Basic Med Sci.

